# Ultrasound Examination of Skin, Fasciae and Subcutaneous Tissue: Optimizing Rehabilitation for Secondary Upper Limb Lymphedema

**DOI:** 10.3390/diagnostics14242824

**Published:** 2024-12-15

**Authors:** Carmelo Pirri, Chiara Ferraretto, Nina Pirri, Lara Bonaldo, Raffaele De Caro, Stefano Masiero, Carla Stecco

**Affiliations:** 1 Department of Neurosciences, Institute of Human Anatomy, University of Padova, 35121 Padova, Italy; rdecaro@unipd.it (R.D.C.); carla.stecco@unipd.it (C.S.); 2 Physical Medicine and Rehabilitation School, University of Padova, Via Nicolò Giustiniani, 2, 35128 Padua, Italy; chiara.ferraretto@gmail.com; 3 Department of Medicine—DIMED, School of Radiology, Radiology Institute, University of Padova, 35122 Padova, Italy; nina_92_@hotmail.it; 4 Department of Neuroscience, Section of Rehabilitation, University of Padova, 35121 Padua, Italy; lara.bonaldo@aopd.veneto.it (L.B.); stef.masiero@unipd.it (S.M.)

**Keywords:** lymphedema, fascia, subcutaneous tissue, ultrasound imaging, echogenicity, thickness

## Abstract

Background: Lymphedema represents a frequent cause of disability for patients undergoing oncological treatments and, being a chronic, non-reversible pathology, requires targeted and continuous rehabilitation treatments. To date, the studies available on the use of ultrasound in patients with lymphedema mainly report descriptive data; therefore, with this study, we wanted to describe in a more objective way the typical ultrasound alterations found in these patients, measuring the thickness of the different superficial structures, and defining subcutis echogenicity. Methods: 14 patients affected by secondary lymphedema of the upper limbs were enrolled in this cross-sectional observational study (12 had breast cancer and 2 with melanoma as their primary diagnosis). All patients were classified as stage II according to the ISL classification. Patients were examined between March and July 2023 with a clinical and an ultrasound evaluation. Ultrasound evaluation was performed following a protocol and took into consideration thickness of the cutis, subcutis, superficial and deep fascia, and subcutis echogenicity. Results: The cutis of the affected limbs was thicker in the distal anterior region of the arm and throughout the anterior region of the forearm. The subcutaneous tissue was thicker in the posterior region of the distal arm and throughout the forearm, including the dorsum of the hand and excluding only the proximal posterior region of the forearm. Fascial structures did not demonstrate statistically significant differences in thickness between pathological and healthy limbs, despite undergoing significant changes from a qualitative point of view (loss of the trilaminar skin appearance and the development of anechoic areas due to fluid accumulation around the hyperechoic adipose lobule). A statistically significant difference in the echogenicity of subcutaneous tissue was found at the distal anterior region of the arm and at the entire anterior forearm. Conclusions: High-resolution ultrasound has been confirmed to be a tool capable of supporting the diagnosis of lymphedema and identifying the most compromised regions of the limb. A tailored rehabilitation plan can be developed based on the non-uniform alterations in subcutaneous tissue, where some areas are affected earlier than others. This compartmentalization should be considered in lymphedema staging and management. Ultrasound may provide early detection of these changes, guiding a more precise therapeutic approach.

## 1. Introduction

Lymphedema affects up to 300 million people worldwide, with approximately 10 million cases in the United States [1,2,3]. Primary lymphedema is rare, with a prevalence of 1–5 per 10,000, while secondary lymphedema, particularly related to cancer treatments, is more common. Studies suggest that one in five women surviving breast cancer may develop lymphedema [1,2,3]. In Italy, around 350,000 people are affected, with 42% of cases being primary, mostly involving the lower limbs, and 58% secondary, often linked to breast cancer and other malignancies. Annually, about 9,000 new cases of secondary lymphedema are diagnosed following breast cancer surgery, highlighting the need for better management and treatment options to reduce the impact on quality of life [4]. The diagnosis of lymphedema is multifaced, involving clinical evaluation, patient history, and physical examination, supplemented by specific diagnostic tools where needed [5]. Traditional methods, such as circumferential measurements, bioimpedance spectroscopy, and water displacement, remain the cornerstone of early detection due to their low cost, accessibility, and ease of use [5]. Bioimpedance spectroscopy, for instance, offers rapid and sensitive detection of extracellular fluid changes, making it a valuable tool for subclinical lymphedema diagnosis [5]. However, its reliance on specialized equipment limits application in resource-constrained settings [5]. More advanced imaging modalities, such as magnetic resonance imaging (MRI) and lymphoscintigraphy, provide detailed anatomical and functional insights, but are associated with higher costs and time requirements as well as limited availability [6]. MRI is particularly suited for characterizing tissue remodeling, such as fibrosis or adipose hypertrophy, but its use is constrained to specialized centers [5] and these tissue changes are only reported when advanced lymphedema occurs. Lymphoscintigraphy, though highly accurate, is less commonly employed due to its complexity and cost. Ultrasound (US) imaging is increasingly being explored as a complementary tool in lymphedema management. It provides the non-invasive visualization of subcutaneous tissue changes, including fibrosis and dermal thickening [5], and might be an option for the early detection of lymphedema/tissue changes in early stages.

Lymphedema is defined as a chronic, progressive condition caused by a disruption in lymphatic transport. The characteristics of edema vary depending on whether it is acute or chronic [6]. Acute lymphedema is usually post-traumatic and temporary, resulting from stasis and the consequent accumulation of fluids in the interstitial space of the skin and subcutaneous tissue [6]. Chronic lymphedema, on the other hand, begins with fluid accumulation in the extracellular matrix due to hyperfiltration or the insufficient resorption of lymphatic fluid [7]. In some cases, when lymphedema persists for at least three months, it is more appropriate to refer to it as “chronic lymphedema” rather than lymphedema, as this term encompasses more complex edema conditions [7]. The volume increase can affect the extremities, entire limbs, and genital area, as well as the trunk, neck, and head [8]. Chronic lymphedema can result from various pathological conditions: primary or secondary lymphedema, venous insufficiency (phlebolymphedema), immobility, edema associated with lipedema, advanced oncological disease, obesity, heart failure, and rare vascular malformations (e.g., Klippel–Trenaunay syndrome). In lymphedema, there is an abnormal accumulation of protein-rich interstitial fluid in the subcutaneous tissue. Persistent lymphatic stasis leads to chronic inflammation, causing the fibrosis of connective tissue with the proliferation and deposition of adipose tissue [9,10,11,12].

Despite fibrotic alterations being a key element used to define the staging and prognosis of lymphedema, the fibrous organization of subcutaneous tissue and whether it varies in lymphedema have not yet been considered. Subcutaneous tissue is divided by the superficial fascia into two distinct compartments, each with unique characteristics: superficial adipose tissue (SAT) and deep adipose tissue (DAT). The superficial fascia is recognized as a specific anatomical structure with distinctive cellular and innervation properties. It is a thin, fibrous membrane that extends continuously throughout the body, composed of irregularly arranged collagen fibers interspersed with numerous elastic fibers [13,14]. This fibrous membrane functions as a supportive scaffold for adipose lobules [13,14]. In recent years, high-resolution ultrasound (US) imaging has been increasingly employed to assess tissue alterations in patients with lymphedema [13,14], as traditional imaging techniques are often costly and time-consuming. US imaging offers several advantages, including low cost, the portability of equipment, and safety during US examination. Specifically for lymphedema, US imaging aids in supporting diagnostic hypotheses and the monitoring of tissue response to decongestive therapy [13,14]. Indeed, physiotherapy (PT) plays a key role in the prevention and management of secondary lymphedema. Techniques such as manual lymphatic drainage, compression therapy, and exercise programs have proven effective in reducing lymphedema incidence and severity by promoting lymphatic circulation and reducing fluid accumulation [15,16,17,18]. Early PT intervention has been shown to significantly lower the risk of secondary lymphedema in patients undergoing axillary lymph node dissection, with evidence reporting a 74% reduction in risk compared to standard care [19]. Exercise programs, including resistance training and aerobic activities, are safe and beneficial, helping to prevent lymphedema onset and improve patient outcomes [15,16]. Despite these benefits, PT remains underutilized due to limited awareness and insufficient resources in many healthcare settings [20]. Early diagnosis and timely physiotherapy interventions are critical for preventing irreversible lymphatic damage and improving long-term outcomes in this patient population [20].

The aim of this study is to analyze, using an US protocol published by Pirri et al. [21,22], the alterations in superficial soft tissues (skin, subcutaneous tissue, and superficial fascia) in patients with chronic lymphedema, in order to derive valuable insights for clinical outpatient assessment and rehabilitative treatment. The current literature lacks data on potential changes in the fascial system in chronic lymphedema. Given the close correlation between fascial structures (particularly the superficial fascia) and the lymphatic system, we explored possible ultrasound-detectable variations in these structures. In this context, we also assessed skin, superficial fascia, deep fascia, and subcutaneous thickness at various levels and regions of the upper limb to identify areas of greater involvement. In addition, the subcutaneous tissue echogenicity was evaluated and compared to describe any alterations. Finally, the study sought to identify correlations between clinical assessment data and US findings.

## 2. Materials and Methods

### 2.1. Study Design

A cross-sectional observational study based on the Strengthening the Reporting of Observational Studies in Epidemiology (STROBE) statement was conducted [23] with the aim of comparing the US thickness of the skin (epidermis + dermis), subcutaneous tissue, superficial fascia, and deep fascia in lymphedematous limbs with those of healthy contralateral limbs. Additionally, changes in subcutaneous echogenicity were analyzed and compared between the affected and healthy limbs. The Helsinki Declaration and human experimentation rules [24] were considered and the Ethics Committee of the University of Padova approved the research. All participants were informed and provided written consent prior to inclusion in the study.

### 2.2. Participants

A total sample of 14 patients with secondary upper limb lymphedema due to oncological conditions were enrolled in this study. These patients were followed through regular check-ups at the specialized lymphedema clinic of the Rehabilitation Unit of Padua Hospital between March and July 2023. The inclusion criteria for participation consisted of some parameters: (1) a diagnosis of secondary lymphedema, typically resulting from cancer treatments such as surgery (e.g., lymph node dissection) or radiation therapy; (2) chronic condition: the lymphedema had to be in a chronic phase, often defined as persisting for at least 6 months. This ensured that acute or transient cases were excluded; (3) lymphedema of stage II or higher; (4) stable condition: candidates had to have stable limb volume, meaning the size of the affected limb did not fluctuate more than 10% over the last two years. This is important for ensuring that US imaging was not confounded by sudden changes in limb size. Patients with lower limb lymphedema; primary lymphedema; lymphedema secondary to venous disease; bilateral lymphedema; active lymphangitis, fever, or recurrent infectious conditions such as erysipelas; male patients; and those with edema related to systemic conditions (e.g., heart, liver, or kidney failure) were excluded.

### 2.3. Rehabilitative and Clinical Examination

**Figure 1:**
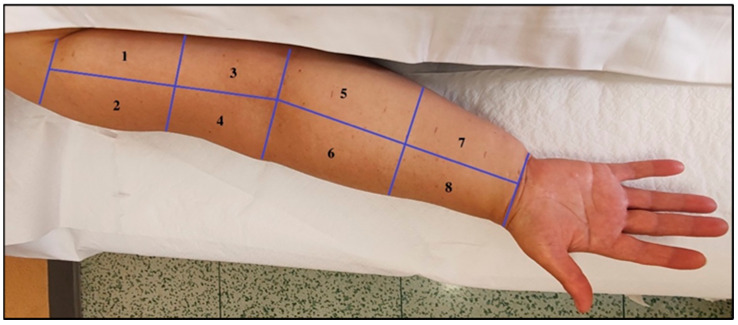
Representation of the upper limb quadrant division: 1—proximal anteromedial arm; 2—proximal anterolateral arm; 3—distal anteromedial arm; 4—distal anterolateral arm; 5—proximal anteromedial forearm; 6—proximal anterolateral forearm; 7—distal anteromedial forearm; 8—distal anterolateral forearm.

The collection of patient history involved identifying relevant risk factors and clinically significant events. Physical examination included gathering anthropometric data, inspecting the affected area, and assessing pliability, tissue texture, and ballotability. Edema quantification was performed using the Edema Pitting Scale. Indirect volumetric measurement of upper limbs was achieved through circumferential measurements, applying the truncated con formula for accuracy. To standardize the assessment of pliability across the entire arm, the upper arm and forearm were divided into quadrants, taking into account the subdivision formed by the adherence between the superficial and deep fascia. the dorsum of the hand and the axillary region were evaluated separately. Table 1 provides the quadrant divisions for the upper arm and forearm, while Figure 1 illustrates the anterior quadrant division, with a mirrored approach for the posterior region.

**Figure 2:**
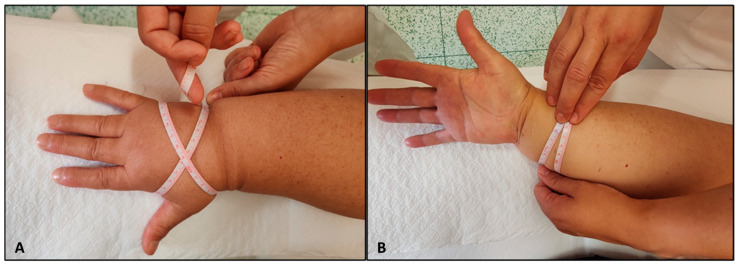
(**A**): Circumferential measurements of the hand were performed using the “figure-of-eight” method, which involves wrapping a millimeter tape in a specific pattern around hand to capture the dimensions accurately. (**B**): For the forearm, circumferential measurements were taken every 5 cm, using a millimeter tape to ensure precision and tissue texture across all measurements.

Pliability was classified as either normal or reduced, depending on whether lymphedema impaired the ability to lift the skin by pinching it between the thumb and forefinger. Reduced pliability could result from fluid accumulation in the subcutaneous tissue and/or dermis, or from fibrotic changes leading to a loss of elasticity in the affected tissues [25,26]. Edema quantification was performed using the Edema Pitting Scale, based on the quadrant division of the upper limb employed in the pliability assessment. The presence of pitting edema was examined in each quadrant and on the dorsum of the hand [26,27]. Ballotability was assessed globally at the level of the arm and forearm by manually pressing the soft tissues and it was categorized as either normal or reduced. Circumferential measurements were taken starting from the wrist crease, which remains visible even in advanced stages of lymphedema. Using a millimeter tape, measurements were recorded every 5 cm up to the axilla. These measurements were consistently performed by the same operator to minimize variability, ensuring that no excessive pressure was applied during the process. For the hand, the “figure-of-eight” measurement method was employed, as depicted in Figure 2 [28].

The volume of the upper limbs was estimated using the truncated cone formula, which allows for an approximation of limb segments based on circumferential measurements [28]. This method provides an accurate estimation by treating each segment of the limb as a conical section, with measurements taken at regular intervals.

### 2.4. Cancer-Associated Lymphedema of the Upper Extremities (CLUE)

The CLUE (Cancer-Associated Lymphedema of the Upper Extremities) scale was used in our study to standardize the clinical assessment of lymphedema, providing a single score that incorporates various aspects of the condition. The scale includes subscales evaluating the reduced visibility of anatomical structures, altered anatomical profiles, tissue changes, and the presence of edema. In the early stages (stage 0, 1, or 2), pitting edema is present due to the predominance of fluid, whereas in advanced stages, tissue fibrosis leads to reduced or absent pitting. Each subscale is scored from 0 to 18 for different segments of the upper limb (arm, forearm, hand), with a maximum total score of 72. On average, patients without lymphedema score lower than those with stage 1 lymphedema and the total score generally increases with disease progression [25].

### 2.5. Ultrasound Examination Measurements

Ultrasound examinations were performed using a high-resolution US machine (Hitachi Avius, Hitachi, Milan, Italy) equipped with a linear L75 probe operating at a frequency range of 5–18 MHz. A physician specializing in physical and rehabilitation medicine with 8 years of experience in the US examination of fasciae (C.P.) carried out the US assessments. The US system operated at a speed of sound of c = 1540 m/s, a standard setting for diagnostic ultrasound machines, and was configured to B-mode with a depth of penetration set at 30 mm. A thick layer of gel was applied to ensure proper contact and to minimize pressure on the skin. The probe was placed as gently as possible to avoid compressing the tissues and was kept perpendicular to the fascial layers to mitigate anisotropy artifacts. The power and overall gain settings of the US machine were carefully standardized and kept consistent across all evaluations to ensure uniformity in image quality and diagnostic accuracy. The short axis was used to provide the most optimal visualization and tracking of anatomical landmarks associated with fascial layers. This orientation allowed for clearer differentiation and continuity of these structures throughout the examination. Identical scanning techniques were used on both the affected and contralateral healthy limb for each patient. During the procedure, the patients lay supine for the examination of the anterior region and prone for the posterior region, with their resting on the examination table. For each level and region analyzed, the protocol described by Pirri et al. [21,22] was followed.

Arm:
  - Anterior region: The patient was positioned supine with the upper limb in neutral position.
    ○ Anterior 1 (Ant 1): The probe was placed in a short-axis view over the proximal half of the arm. The median nerve and brachial artery were located medially, between the brachialis and triceps muscles.
    ○ Anterior 2 (Ant 2): The probe was moved downward, just above the cubital fossa, following the median nerve and brachial artery. The brachialis muscle can be visualized deep into the biceps muscle.
    ○ Axilla: The patient is positioned with the arm elevated on the bed to optimally expose the axillary region. The probe was placed in a long-axis view over the anterior pillar of the axilla, precisely consisting of the pectoralis major muscle.
  - Posterior region: The patient was positioned prone, with the upper limb in neutral position.
    ○ Posterior 1 (Post 1): The probe was placed in a short-axis view over the proximal half of the posterior arm, allowing the visualization of the three heads of the triceps muscle. The median, ulnar, and radial nerves could also be seen nearby.
    ○ Posterior 2 (Post 2): The probe was moved downwards, following anatomical landmarks to just above the elbow.
Forearm:
  - Anterior region: The patient was supine with the upper limb in a neutral position and the forearm supinated.
    ○ Anterior 1 (Ant 1): The probe was placed in a short-axis view on the proximal anterior forearm. The pronator teres was located laterally to the flexor carpi radialis (FCR) and flexor digitorum superficialis (FDS). The median nerve could be seen between the two heads of the pronator teres.
    ○ Anterior 2 (Ant 2): The probe was moved downwards to the distal anterior forearm. The flexor digitorum profundus (FDP) was visible deep into the FDS, flexor carpi ulnaris (FCU), and palmaris longus. These structures lie above the interousseus membrane.
  - Posterior region: The patient was prone with the upper limb in a neutral position and the forearm pronated.
    ○ Posterior 1 (Post 1): The probe was placed in a short-axis view on the proximal posterior forearm, just below the elbow. At this level, the extensor digitorum (ED) was located between the extensor carpi radialis brevis (ECRB) and extensor digitorum brevis (EDB), above the supinator muscle. The brachioradialis muscle could be observed medially, along with the radial artery and nerve branches.
    ○ Posterior 2 (Post 2): the probe was moved downwards over the distal posterior forearm. The ED became smaller while EDB increased in size. The abductor pollicis longus (APL) was seen near the radius, while the extensor pollicis longus (EPL) was positioned near the ulna. The extensor carpi ulnaris (ECU), located most medially, lay above the ulna, medial to the EDB, EPL, and APL. The extensor pollicis brevis and extensor indicis were also visualized.
    ○ Dorsum of hand: The probe was positioned on the dorsum of the hand to assess the skin and subcutaneous tissue, ensuring optimal contact while minimizing pressure to avoid compressing the soft tissues and altering the accuracy of the measurements.

After identifying the relevant bony, muscular, nerve, and tendinous landmarks for each point as described earlier, the image depth was adjusted to optimize the visualization of the superficial layers. The focus was typically positioned between 0.5 and 2 cm, ensuring clear imaging within the first 3 cm. However, for the posterior region of the arm, the focus was placed at 3 cm to accommodate the deeper structures in that area. This approach ensured consistent clarity across all regions examined.

### 2.6. Ultrasound Image Analysis

US images were captured and stored at the conclusion of each evaluation. Subsequent image analysis was conducted independently by a second operator with extensive training in ultrasonographic image interpretation. This second operator was blinded to the clinical status of the limb (healthy vs. affected) to eliminate potential bias in the assessment of tissue characteristics. The blinding ensured that measurements of thickness and structural changes were based on the objective analysis of the US images. This dual-operator methodology was designed to minimize observer bias and enhance the reliability of the findings. For each level and region, the thickness of the skin, subcutaneous tissue, superficial fascia, and deep fascia were measured in millimeters using ImageJ software (version 1.54f, accessible online: https://imagej.nih.gov/ij/, accessed on 7 October 2023). To minimize the impact of potential thickness variation, each image was divided into three regions. Three points of interest were measured for each structure within the image; in each of them, three points representing the best visibility were measured and averaged for statistical analysis. The skin thickness was measured from the most superficial hyperechoic interface to the dermo-hypodermal junction. Subcutaneous thickness was assessed from the dermo-hypodermal junction to the deep fascia, excluding the deep fascia itself. The superficial fascia thickness was measured at the hyperechoic waved structure that bisects the subcutaneous tissue, while the deep fascia thickness was determined at the hyperechoic structure between the subcutaneous tissue and the underlying muscle. To quantitatively evaluate changes in the microstructure of lymphedematous tissue, a region of interest (ROI) corresponding to the entire subcutaneous tissue was manually delineated for each scan. The average pixel values within the ROI were used to assess tissue echogenicity. Echogenicity was determined by obtaining the mean grayscale value, ranging from = 0 (black) to 255 (white), using ImageJ software. Each image was digitized and analyzed using the standard grayscale histogram function. Pixel intensity was measured for the subcutaneous tissue in both the lymphedematous limb and the healthy contralateral limb for all patients.

### 2.7. Statistical Analysis

Statistical analysis was performed using GraphPad PRISM 8.4.2 (GraphPad Software Inc., San Diego, CA, USA), with *p* < 0.05 considered the threshold for statistical significance. The effect size was calculated with G power 3.1 (Unversität Düsseldorf, Psychology) and interpreted according to Cohen’s kappa as small (d = 20), medium (d = 0.50), and large (d = 0.80) [29]. In our previous study [17,18], the effect size for the superficial fascia of the arm and forearm was d = 1.2, corroborated by additional research [30]. The statistical parameters included an error probability (α error prob) of 0.05 and a statistical power (1-β err prob) of 0.95, leading to a required sample size of 10 participants. However, we were able to expand our cohort, ultimately including 14 patients in the study group. This sample size allowed for a more robust analysis and improved the validity of our findings. Normality was assessed using the Kolmogorov–Smirnov test. Descriptive statistics, including central tendency measures and dispersions, were calculated using mean and standard deviation (SD) to describe parametric data. Comparative analysis across different levels was conducted using a one-way ANOVA, followed by Tukey’s post hoc test for multiple comparisons. Comparisons between pathological and healthy limbs were performed using a paired Student’s *t*-test. Finally, Pearson’s correlation test was employed to evaluate the relationship between total CLUE score, lymphedema duration, limb volume, and subcutaneous tissue thickness and echogenicity for both limbs of the patients.

## 3. Results

### 3.1. Descriptive Data

The enrolled patients were aged between 44 and 74 years old, with an average age of 60 years. The mean BMI was 26.83 ± 4.65 kg/m^2^, with five patients categorized as normal weight, four as overweight, and five classed as obese. Twelve of the participants had been diagnosed with breast carcinoma, while two had a diagnosis of scapular melanoma. At the time of evaluation, the time since their first surgery ranged from 3 to 21 years, with 71.4% of patients having undergone additional surgical procedures following the initial operations. In terms of surgical interventions, 50% of the patients had undergone a mastectomy, 35.7% had a quadrantectomy, and 14.3% had undergone resection of a scapular melanoma with a wider margin excision. Additionally, 92.8% of the patients had an axillary lymph node dissection, while only 7.1% had sentinel lymph node removal. Regarding chemotherapy, 92.8% of the patients required neoadjuvant or adjuvant therapy. Notably, 74.3% received taxane-based chemotherapy. Moreover, 50% of the cases required additional hormonal therapy. Radiotherapy was administered to 71.4% of the patients, with treatment targeting the thoraco-mammary region. in 42.8% of these cases, axillary irradiation was also included, while 28.6% received irradiation to both the axillary and supraclavicular regions. All participants had stage II lymphedema, as classified by the International Society of Lymphology. Of the 14 patients, 7 were affected in the right upper limb and 7 in the left upper limb. The onset of lymphedema occurred, on average, 14 months after the first surgery, with a range of 1 month to 4 years. At the time of evaluation, lymphedema had been present for an average of 4 years. Five patients reported episodes of lymphangitis since the onset of lymphedema. Table 2 outlines the characteristics of the study population.

Complex decongestive therapy, which includes manual lymphatic drainage combined with multilayer bandaging and physical activity while wearing the bandage, was regularly performed by 13 patients (92.8%). These treatments are typically conducted as part of an intensive cycle prior to the renewal of a custom-made compression garment prescription or when there is clinical deterioration. After the custom compression sleeve is fabricated, decongestive therapy is continued weekly or bi-weekly to maintain the results achieved. At the time of assessment for this study, all 14 patients (100%) were using a custom-made flat knit compression sleeve, class II compression, extending from the axilla to the wrist. Additionally, 10 patients (71.4%) required a separate glove (Table 3).

During clinical/rehabilitative evaluation, pliability was assessed in each quadrant of both the anterior and posterior regions of the upper limbs. The healthy limb served as the reference for normal pliability in each patient. Table 4 presents the number of lymphedema patients showing reduced pliability in different quadrants of the limbs. For the arm, the distal posteromedial region was impaired in all patients, whereas for the forearm, the entire anteromedial region (both proximal and distal) exhibited reduced pliability, along with the proximal posteromedial region (92.8%).

In addition to assessing pliability, edema was evaluated using the Edema Pitting Scale. Table 5 presents the average values obtained from this scale, broken down by quadrants of the upper limb.

Ballotability was reduced in the forearm in 10 patients (71.4%), while no patients exhibited reduced ballotability in the arm. Table 6 provides the average score obtained from the administration of the CLUE scale across the study population.

Indirect volumetric measurements of the upper limbs revealed an average volume of 2188.5 mL for the affected limb, compared to 1844.9 mL for the healthy limb. The average percentage difference in volume between the affected and healthy limbs was 18.95% (Table 7).

### 3.2. Qualitative Analysis of Ultrasound Assessments

**Figure 3:**
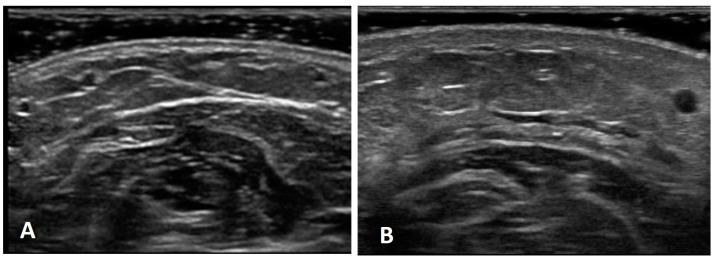
(**A**) Normal US image of the healthy limb, showing the preserved trilaminar structure of the skin, the normal structure of subcutaneous tissue with superficial fascia, and normal deep fascia. (**B**) US appearance of the limb affected by chronic lymphedema, demonstrating a preserved trilaminar structure of the skin with increased thickness compared to the healthy limb. The subcutaneous tissue exhibits hyperecheoic regions, while the superficial fascia remains well defined, despite the tissue change. The deep fascia is identifiable. These findings reflected the structural remodeling characteristics of the different stages of chronic lymphedema.

The ultrasound examination of the upper limbs affected by lymphedema demonstrated a variety of distinct patterns in the superficial soft tissue. These patterns, which involved both skin and subcutaneous tissue with fasciae, differed based on the stage and progression of chronic lymphedema. Among those with chronic lymphedema, the skin in cases with a shorter duration retained its trilaminar structure, while the subcutaneous tissue showed hypoechoic areas with superficial fascia. This structure often appeared disrupted and discontinuous when compared to the healthy side, despite maintaining its thickness. However, as the disease advanced, new patterns emerged, including the loss of the trilaminar skin appearance and the development of anechoic areas due to fluid accumulation around the hyperechoic adipose lobules. Despite these variations, the superficial fascia and deep fascia often remained intact, highlighting its resilience amid the diverse patterns of tissue change caused by lymphedema (Figure 3).

### 3.3. Ultrasound Thickness Measurements

#### 3.3.1. Skin: Epidermis and Dermis

**Figure 4:**
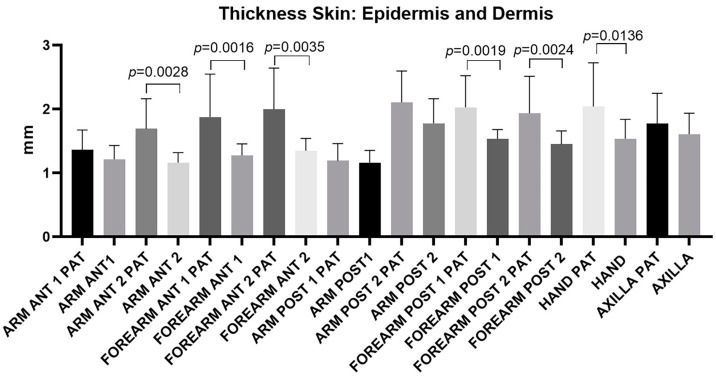
Ultrasound measurements of skin thickness (epidermis and dermis) taken from various regions and levels of the affected upper limb (PAT) and the healthy upper limb. The figure highlights the levels/regions where a statistically significant difference in thickness was found between the two limbs. These significant differences indicate a marked increase in skin thickness in the pathological limb, underscoring the impact of lymphedema on tissue structure across specific levels/regions of the upper limb.

Figure 4 depicts the distribution of skin thickness across various regions of the arm, forearm, hand, and axilla. On average, the skin thickness is greater in the limb affected by lymphedema (“PAT”) compared to the healthy limb. Statistically significant differences in skin thickness were observed in the regions identified as Anterior arm 2: *p* = 0.0028, Anterior forearm 1: *p* = 0.0016, Anterior forearm 2: *p* = 0.0035, Posterior forearm 1: *p* = 0.0019, Posterior forearm 2: *p* = 0.0024, and the dorsum of the hand: *p* = 0.0136, highlighted in Table 8. These data underscore the localized impact of lymphedema on tissue thickness.

#### 3.3.2. Subcutaneous Tissue

**Figure 5:**
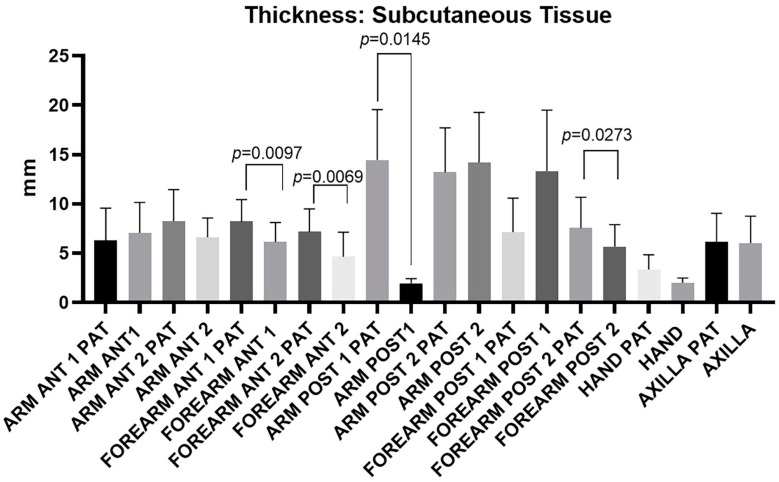
Ultrasound measurements of subcutaneous tissue thickness taken from different regions and levels of both the affected upper limb (PAT) and the healthy limb. The figure highlights the levels/regions where statistically significant differences in thickness were observed between the pathological and healthy limbs. These findings underscore the localized nature of tissue alterations in lymphedema and their varying impact across different anatomical regions.

Figure 5 illustrates the distribution of subcutaneous tissue thickness across various levels/regions of the arm, forearm, hand, and axillary region. Interestingly, not all regions of the affected upper limb show an average increase in subcutaneous tissue thickness; statistically significant differences in thickness were identified in levels/regions labeled as Anterior forearm 1: *p* = 0.0097, Anterior forearm 2: *p* = 0.0069, Posterior arm 1: *p* = 0.0145, and Posterior forearm 2: *p* = 0.273, which are highlighted in green in Table 9. These findings indicated that lymphedema-related tissue changes are localized and not uniform across the entire limb.

#### 3.3.3. Superficial Fascia

**Figure 6:**
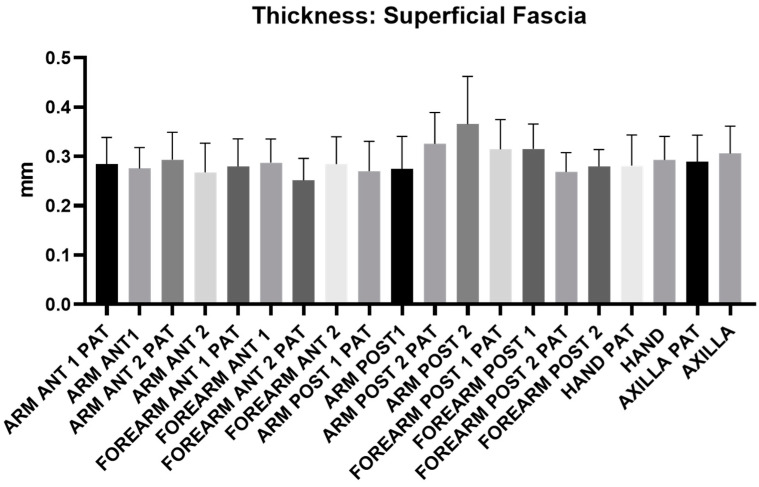
Ultrasound measurements of the superficial fascia thickness taken across different regions and levels of the affected upper limb (PAT) and the healthy limb. No statistically significant differences in thickness were found between the pathological and healthy limbs in any of the levels/regions.

Figure 6 illustrates the distribution of superficial fascia thickness across different levels/regions of the arm, forearm, hand, and axillary region. No level/region showed a statistically significant difference in thickness between the affected and healthy limbs. The average thickness of the superficial fascia was 0.29 mm for both arm and forearm, with similar values observed between the pathological and healthy limbs (Table 10). These findings indicate that the superficial fascia’s thickness remains consistent despite the presence of lymphedema.

#### 3.3.4. Deep Fascia

**Figure 7:**
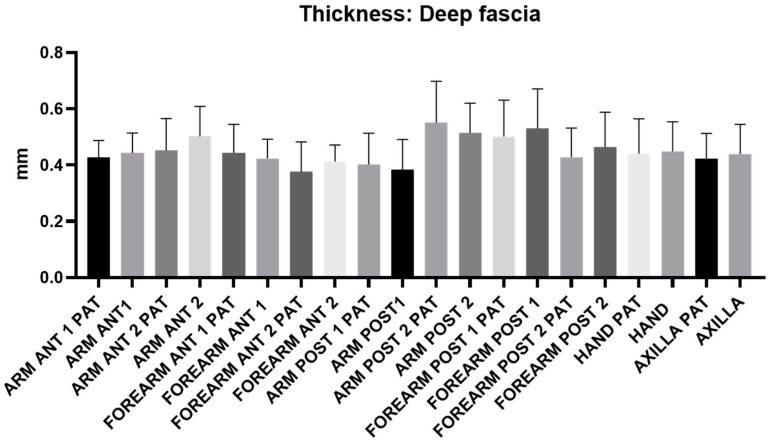
Ultrasound measurements of deep fascia thickness were conducted across various regions and levels of both the affected upper limb (PAT) and the healthy limb. No statistically significant differences in thickness were found between the two limbs.

Figure 7 illustrates the distribution of deep fascia thickness across various levels and regions of the arm, forearm, hand, and axillary region. No statistically significant differences in thickness were observed in any levels/regions. The average thickness of the deep fascia was 0.45 mm for both the arm and forearm, with similar values recorded between the affected and healthy limbs (Table 11).

### 3.4. Echogenicity Measurements

**Figure 8:**
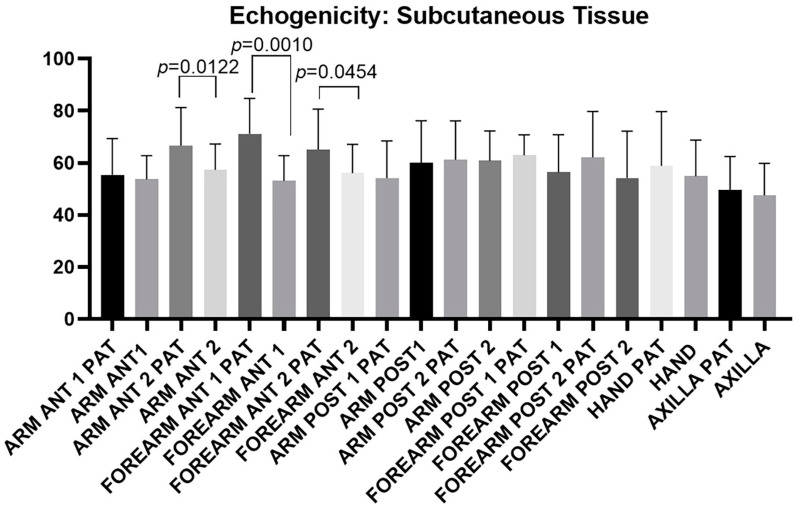
Echogenicity of the subcutaneous tissue measured across different regions and levels of both the affected upper limb (PAT) and the healthy limb. The figure highlights the regions and levels where statistically significant differences in echogenicity were observed between pathological and healthy limbs. These differences indicate localized changes in tissue composition due to lymphedema, particularly in regions/levels where increased echogenicity was detected.

Figure 8 illustrates the echogenicity of subcutaneous tissue across various regions of the arm, forearm, hand, and axilla. Notably, not all regions of the affected upper limb showed a uniform increase in subcutaneous tissue echogenicity. Statistically significant increases in echogenicity were observed in the regions identified as Ant 2 arm: *p* = 0.0122, Ant 1 forearm: *p* = 0.001, and Ant 2 forearm: *p* = 0.0454, highlighted in green in Table 12. This increase suggests localized tissue changes associated with chronic lymphedema in specific areas of the upper limb.

### 3.5. Correlations

Statistically significant correlations were found between the total CLUE score and the proximal posterior region (Post 1) of the arm, as well as the entire posterior region of the forearm, including the dorsum of the hand (Table 13). As the CLUE score increases, so does the echogenicity in these posterior regions of the upper limb. These findings align with the fact that a higher CLUE score typically indicates a more advanced stage of the disease and the posterior region of the upper limb tends to be affected in the later stages of lymphedema progression. This suggests that as lymphedema progresses, marked by higher CLUE scores, subcutaneous tissue undergoes structural changes, leading to a higher echogenic profile. This increased echogenicity is likely reflective of greater fibrosis or fluid accumulation, which are hallmarks of more advanced stages of the disease.

A statistically significant correlation was found between the volume of the limb affected by lymphedema and the thickness of the subcutaneous tissue in the distal posterior region of the arm and the entire posterior region of the forearm (Table 14). These posterior regions of the upper limb appear to play a key role in contributing to a greater increase in the overall volume of the limb. This finding suggests that as the disease progresses, the posterior regions of the limb may be more prone to substantial subcutaneous changes, thereby driving the increase in limb volume commonly observed in advanced stages of lymphedema. This suggests that the subcutaneous tissue thickening may be a primary contributor to the overall volumetric increase observed in lymphedema-affected limbs.

Lastly, the duration of lymphedema shows a positive correlation with the thickness of the subcutaneous tissue in the distal anterior region of the forearm and a negative correlation with echogenicity in the distal posterior region of the forearm (Table 15). These findings suggest that as the duration of lymphedema increases, there is a corresponding thickening of the subcutaneous tissue in the anterior region, while echogenicity may be related to the delayed accumulation of lymph in the posterior forearm compared to the anterior region.

### 3.6. Intra-Rater Reliability

Additionally, the intra-rater reliability was reported to be consistently high, ranging from good to excellent. The results for the skin, superficial fascia, deep fascia, and subcutaneous tissue thickness measurements are reported in Table 16. These values underscore the high level of consistency and reproducibility of the measurements across both upper limbs.

## 4. Discussion

Based on our current knowledge, this study may be stated as the first study detailing skin, fasciae, and subcutaneous tissue parameters in patients with chronic secondary lymphedema based on clinical/rehabilitative and US examinations. As far as the authors of the present study are aware, most studies on the use of US examination in patients with chronic lymphedema have primarily provided descriptive data. In contrast, our study aimed to offer an objective assessment of the typical US findings in these patients by quantifying the thickness of various superficial structures and determining the echogenicity of the subcutaneous tissue. This approach allows for a more precise understanding of the tissue changes that occur in lymphedema, beyond mere qualitative descriptions, thereby contributing to a deeper understanding of the pathology through measurable parameters.

The study’s primary aim was to study the skin, superficial fascia, deep fascia, and subcutaneous thickness in this type of patient. The findings showed a statistically significant increase in average skin thickness across the entire forearm (both anterior and posterior) in upper limbs affected by lymphedema compared to healthy limbs (Figure 4, Table 8). Similarly, increased skin thickness was observed in the dorsum of the hand and the distal region of arm. These results suggest a consistent pattern of tissue thickening in regions impacted by lymphedema, highlighting the extent of structural changes in the skin across various regions of the upper limbs. Regarding the quantification of increased skin thickness, the available literature data are sometimes outdated and often present thickness as an average valued limited to the forearm [30,31].

In addition, the study’s findings indicated a broader increase in subcutaneous tissue thickness on the pathological side, including the distal posterior arm and the entire forearm, as well as the dorsum of the hand, with the proximal posterior level/region being the only exception (Figure 5, Table 9). The available literature on subcutaneous tissue thickness measured by US examination is sparse. For instance, Devoogdt et al. [32] reported that increased subcutaneous tissue thickness was only observed in the distal posterior region of the arm, with measurements showing 11.76 mm in the affected limb versus 9.95 mm in the healthy limb, 12 months after surgery. It is unlikely that this increased subcutaneous tissue thickness is solely due to fluid accumulation. Excessive growth of fibrous and adipose tissue can also contribute to this thickening. When interstitial fluid increases, there is a corresponding increase in extracellular matrix production by fibroblasts, which leads to greater water entrapment in the interstitium. Over time, this can result in fibrosis, as described by Mellor et al. [30]. These processes underline the complexity of tissue changes in lymphedema beyond mere fluid retention.

No statistically significant differences in the thickness of the superficial (Figure 6, Table 10) and deep (Figure 7, Table 11) fasciae were observed between the affected and healthy upper limbs. However, instead of a change in thickness, qualitative alterations were noted, particularly in the superficial fascia. In chronic stages of lymphedema, this structure often appeared disrupted and discontinuous when compared to the healthy side, despite maintaining its thickness. The close relationship between the superficial lymphatic system and the superficial fascia has several clinical implications. It is well known that initial lymphatic vessels rely on the support of the extracellular matrix, maintained by anchoring filaments, to preserve their patency. Therefore, any disruption to the superficial fascia and its anchoring fibers/retinacula cutis could further impair lymphatic drainage. Additionally, the close association between lymphatic vessels and fibrous septa/retinacula cutis in the superficial adipose tissue suggests that the elasticity and organization of connective tissue may influence lymphatic transport from the dermal plexus to deeper lymphatic vessels/collectors. This could either enhance or hinder lymphatic function, as fibrosis and decreased elasticity in the subcutaneous tissue are likely to reduce the lymphatic drainage capacity [33]. Given that fascial structures are easily identifiable in US imaging, any quantitative and, especially, qualitative alterations should be carefully evaluated in lymphedema patients. Despite no difference in the fascial thickness between the two upper limbs being highlighted in this study, the superficial fasciae in this population were thinner with respect to the data already published in a healthy population [21,22,30]. These findings suggest that the patients with chronic lymphedema could already have an altered superficial fascia that can induce lymphedema. This anatomical understanding further highlights the importance of thoroughly assessing fascial alterations during US examinations.

Several studies in the literature have described changes in the echogenicity of the dermo-epidermal complex and subcutaneous tissue in upper limbs affected by lymphedema [26,31,34]. However, to our knowledge, this is the first study to systematically evaluate subcutaneous tissue echogenicity following a standardized protocol and to quantify these changes compared to healthy limbs. Van der Veen et al. [35] reported no significant differences in echogenicity between limbs affected by lymphedema and healthy limbs. In our study, we observed a statistically significant increase in subcutaneous tissue echogenicity in the distal anterior region of the arm (Ant 2 arm) and throughout the anterior forearm compared to the healthy limb (Figure 8, Table 12). These findings likely reflect the typical characteristics of chronic lymphedema, including tissue remodeling and persistent fluid retention. The hyperechoic areas identified on US are interpreted as indicators of increased fluid accumulation and early trophic changes in the subcutaneous tissue, rather than sclerotic alterations. Notably, none of the patients included in the study exhibited clinical or imaging evidence of advanced fibrosis. This suggests that while US changes are prominent in chronic lymphedema, their interpretation must consider the absence or presence of fibrosis to better inform disease management. Conversely, the absence of significant echogenic differences may be due to the fact that lymph fluid has an echogenicity similar to that of adipose tissue. Thus, even though subcutaneous tissue thickness may increase in most regions of the forearm, qualitative changes might not be reflected in quantitative echogenicity analysis alone, where no alterations might be detected. The dermis also showed changes in echogenicity, accompanied by increased thickness, though accurately delineating a region of interest (ROI) for precise echogenicity measurement is challenging. Therefore, dermal echogenicity was not assessed in this study. Similar to the subcutaneous tissue, early-stage dermal backflow may result in an echogenicity resembling normal dermal tissue, with the most reliable indicator being an increase in skin thickness [36]. It is also worth noting that a substantial increase in water content, especially in intermediate stages, can cause collagen bundles in the dermis to separate, leading to a reduction in echogenicity.

As noted by other authors [26,30], our study confirmed that the medial antecubital region, both above and below the elbow, is the most affected area in lymphedema, detectable clinically and via US examination. This region contains the epitrochlear lymph nodes, which drain lymph from the last three fingers and medial forearm toward the axillary nodes [33]. After axillary lymph node dissection, lymphatic dysfunction in this area is plausible due to disrupted downstream pathways. As collateral circulation fails, lymphedema often begins here, with chronic changes such as increased echogenicity (indicating fibro-adipose replacement) being more prominent. By contrast, the distal forearm and hand, influenced by gravity, tend to accumulate free lymph fluid, leading to increased thickness without necessarily altering echogenicity. The upper arm showed no significant fibrotic changes, likely due to Mascagni’s cephalic pathway, the only drainage route bypassing the axillary nodes [37].

Our study revealed a significant correlation between the total CLUE score and subcutaneous echogenicity (Table 13), as well as between limb volume and subcutaneous thickness (Table 14), particularly in the distal posterior arm and entire posterior forearm. These findings suggest that higher CLUE scores and larger limb volumes indicate more advanced lymphedema, with posterior regions becoming involved later in the disease, after anterior regions are fully affected. This highlights the limitations of the ISL staging system, as it does not account for regional progression within a single patient. All patients were classified as stage II, yet the upper limb regions did not show uniform changes. A more nuanced staging system reflecting these regional differences, alongside variability in US findings, CLUE scores, and volumetric classifications, would enhance lymphedema assessment.

### Limitations of Study and Future Perspectives

Firstly, the sample size was relatively small, which may limit the generalizability of the findings to a broader population of patients with chronic secondary lymphedema. Larger studies are needed to validate these results and better understand the variability in tissue changes across different patient populations. Secondly, the cross-sectional design of the study precludes the ability to assess long-term progression and treatment outcomes. Additionally, the accuracy of the US examination of skin, subcutaneous tissue, and fascia morphology is highly dependent on both the expertise of the sonographer and the correct calibration of the US equipment. These factors introduce variability and potential bias, emphasizing the need for standardized protocols, as used in this study, and more robust studies to enhance the reliability of findings and their clinical application. Moreover, the exclusion of conditions associated with sudden changes in limb size, such as acute infections or rapid edema fluctuations, was necessary to avoid confounding the imaging results. However, this exclusion also limits the generalizability of the findings to patients with such conditions. The lack of validated scales for assessing tissue texture, pliability, and ballotability is a limitation of this study. Tissue texture descriptors were adapted from the CLUE scale [25], while pliability and ballotability were qualitatively assessed and categorized as normal or reduced based on clinical experience. Although these terms are widely accepted in clinical practice, the absence of standardized, validated measures may impact reproducibility and comparability. Moreover, this study acknowledges the potential variability of tape measurements due to operator skill. Tape is less reliable than water volumetry, the reference standard for upper extremity lymphedema [38]. Evidence supports the use of a 1 cm wide tape and standardized protocol (e.g., 4 cm intervals) to improve reliability [39]. Lack of standardization may introduce bias, emphasizing the need for training and validated methods [39]. Future research should prioritize the training and validation of measurement protocols to improve reliability.

Future lymphedema classifications could greatly benefit from incorporating US examination, as discussed in this study. A more precise staging system would enable a more tailored rehabilitative approach, adapted to the specific characteristics of each patient. This would allow complex decongestive therapy to be targeted differently in regions/levels with higher fluid content compared to those undergoing fibrotic tissue remodeling. Considering the importance of fascial structures and their close relationship with the lymphatic system, future studies should involve larger populations and include comparisons with healthy individuals. This would allow for a more objective evaluation of potential quantitative changes in these structures, in addition to the qualitative changes already described.

US examination holds promise as a predictive tool in lymphedema management by identifying early tissue changes such as subcutaneous thickening, echogenicity variations, and fibrosis. These markers could enable clinicians to stratify patients, prioritize those likely to benefit most from CDT, and refine treatment pathways. Future studies should focus on developing criteria for predicting fibrosis and lymphedema progression, allowing for more personalized and efficient care while enhancing clinical outcomes.

## 5. Conclusions

In conclusion, when evaluating a patient with chronic secondary lymphedema of the upper limb, particular attention should be given to the medial antecubital region, both clinically and via US examination, even in the early stages of the disease. This region appears to be especially susceptible to lymphatic dysfunction and may be the first to exhibit tissue changes characteristic of lymphedema. In contrast, the posterior region of the upper limb tends to be affected later, once anteromedial region is already compromised from a lymphatic standpoint. While US examination is not irreplaceable, it could add value to the assessment of lymphedema. It enables the visualization of superficial soft tissue changes across different levels and regions of the upper limb, with both qualitative and quantitative detail, all in a cost- and time-efficient manner. Once the most affected regions are identified, a personalized rehabilitation plan can be developed, including targeted manual techniques and the prescription of custom compression garments, such as the introduction of pelotes in specific regions. US examination also allows for monitoring disease progression following intensive treatment or during maintenance therapy. Looking forward, there is a need to develop a lymphedema staging system that accounts for the varying progression of the disease across different regions and levels of the limb, with a particular focus on the fascial system. Such a system would enable a more nuanced and individualized physical therapy approach to both diagnosis and treatment.

## Figures and Tables

**Table 1:** Subdivision into quadrants of the upper limb, used during rehabilitative–clinical examination.

Anterior Region	Posterior Region
Anterior medial proximal	Posterior medial proximal
Anterior lateral proximal	Posterior lateral proximal
Anterior medial distal	Posterior medial distal
Anterior lateral distal	Posterior lateral distal

**Table 2:** Characteristics of the patients included in the study.

Descriptive Variable	Data
Age (mean ± SD)	60.43 ± 8.74 years old
BMI (mean ± SD)	26.83 ± 4.65 kg/m^2^
	Normal weight: 5 patients (35.7%) Overweight: 4 patients (28.6%) Obese (class I): 5 patients (35.7%)
Oncological diagnosis	Breast cancer: 12 patients (85.7%) Melanoma of scapular region: 2 (14.3%)
Type of surgical intervention	Mastectomy: 7 patients (50%) Quadrantectomy: 5 patients (35.7%) Melanoma resection with wider surgical margins: 2 patients (14.3%) Axillary lymph node resection: 13 patients (92.8%) Subsequent re-operation in later years: 10 patients (71.4%)
Chemotherapy treatment (CT)	13 patients (92.8%):
	Neoadjuvant chemotherapy: 10 patients (71.4%) Taxane-based CT: 9 patients (64.3%) Adjuvant hormone therapy: 7 patients (50%) Adjuvant hormone therapy only: 2 patients (14.35)
Radiotherapy treatment (RT)	Thoraco-mammary RT: 10 patients (71.4%) Thoraco-mammary and axillary RT: 6 patients (42.8%) Thoraco-mammary, axillary and supraclavicular RT: 4 patients (28.6%)
Previous lymphangitis episodes	5 patients (35.7%)

**Table 3:** Type of treatment in patients examined.

Treatment	Data
Manual lymphatic drainage and multi-layer bandaging	13 (92.8%)
Custom-made compression sleeve	14 patients (100%) wore a custom-made flat-knit sleeve extending from the axilla to the wrist, class II compression. 10 patients (71.4%) used a glove with separate fingers in addition to the sleeve.

**Table 4:** Percentage of patients with reduced pliability in different quadrants of the upper limb affected by lymphedema.

Region	Quadrant	Quadrant	Quadrant	Quadrant	Quadrant
Arm Anterior	Ant.-med. Prox.: 0 (0%)	Ant.-med. Dist.: 12 (85.7%)	Ant.-lat. Prox.: 0 (0%)	Ant.-lat. Dist.: 8 (57.1%)	Ant. Axilla: 1 (7.1%)
Arm Posterior	Post.-med. Prox.: 1 (7.1%)	Post.-med. Dist.: 14 (100%)	Post.-lat. Prox.: 1 (7.1%)	Post.-lat. Dist.: 10 (71.4%)	Post. Axilla: 4 (28.6%)
Forearm Anterior	Ant.-med. Prox.: 14 (100%)	Ant.-lat. Dist.: 14 (100%)	Ant.-lat. Prox.: 9 (64.3%)	Ant.-lat. Dist.: 12 (85.7%)	-
Forearm Posterior	Post.-med. Prox.: 13 (92.8%)	Post.-med. Dist.: 12 (85.7%)	Post.-lat. Prox.: 12 (85.7%)	Post.-lat. Dist.: 12 (85.7%)	Dorsum hand: 5 (35.7%)

**Table 5:** Average score of the Edema Pitting Scale measured across different quadrants of the upper limb affected by lymphedema.

Region	Quadrant	Quadrant	Quadrant	Quadrant	Quadrant
Arm Anterior	Ant.-med. Prox.: 1+	Ant.-med. Dist.: 2+	Ant.-lat. Prox.: 1+	Ant.-lat. Dist.: 2+	Ant. Axilla: 1+
Arm Posterior	Post.-med. Prox.: 1+	Post.-med. Dist.: 3+	Post.-lat. Prox.: 1+	Post.-lat. Dist.: 2+	Post. Axilla: 1+
Forearm Anterior	Ant.-med. Prox.: 3+	Ant.-lat. Dist.: 3+	Ant.-lat. Prox.: 3+	Ant.-lat. Dist.: 3+	-
Forearm Posterior	Post.-med. Prox.: 3+	Post.-med. Dist.: 3+	Post.-lat. Prox.: 3+	Post.-lat. Dist.: 3+	Dorsum hand: 2+

**Table 6:** Score obtained from the CLUE scale, expressed as the average score and divided into the various subscales.

Reduced Visibility of Anatomical Structures	Deviation from Normal Anatomical Profiles	Tissue Alteration (Consistency)	Presence of Edema	Total Score
12	10	8	13	43

**Table 7:** Volumetric difference between the limb affected by lymphedema and the contralateral healthy limb, expressed in ml (mean and standard deviation) and classification of patients based on the severity of lymphedema according to the percentage volume difference between the two limbs.

**Volume of limb affected by lymphedema**	2188.5 (±497.3) mL
**Volume of contralateral healthy limb**	1844.9 (±358.5) mL
**Average percentage difference between the two limbs**	18.95%
**Severity of lymphedema**	1 (7.1%) no lymphedema 9 (64.3%) minimal lymphedema 3 (21.4%) moderate lymphedema 1 (7.1%) severe lymphedema

**Table 8:** Ultrasound measurements of skin thickness in the arm, expressed in millimeters. The segment highlighted in green indicates where a statistically significant difference in thickness was observed between the affected and healthy limb (*p* < 0.05). These highlighted values emphasize the specific regions most impacted by lymphedema-related changes in skin structure. SD: standard deviation. CI: confidence interval. The segment highlighted in green indicates a statistically significant difference in echogenicity between the affected and healthy limbs.

Skin: Epidermis and Dermis			
Arm	Mean (SD) [95% CI]	Forearm	Mean (SD) [95% CI]
Ant 1 PAT	1.368 (0.31) [95% CI: 1.192–1.544]	Ant 1 PAT	1.876 (0.67) [95% CI: 1.488–2.265]
Ant 1	1.214 (0.22) [95% CI: 1.087–1.340]	Ant 1	1.275 (0.18) [95% CI: 1.170–1.380]
Ant 2 PAT	1.692 (0.47) [95% CI: 1.420–1.965]	Ant 2 PAT	1.999 (0.64) [95% CI: 1.627–2.372]
Ant 2	1.163 (0.20) [95% CI: 1.073–1.253]	Ant 2	1.352 (0.20) [95% CI: 1.243–1.462]
Post 1 PAT	1.191 (0.30) [95% CI: 1.035–1.348]	Post 1 PAT	2.029 (0.50) [95% CI: 1.743–2.315]
Post 1	1.163 (0.20) [95% CI: 1.035–1.290]	Post 1	1.534 (0.15) [95% CI: 1.448–1.619]
Post 2 PAT	2.106 (0.50) [95% CI: 1.822–2.389]	Post 2 PAT	1.935 (0.60) [95% CI: 1.601–2.269]
Post 2	1.779 (0.40) [95% CI: 1.556–2.001]	Post 2	1.452 (0.20) [95% CI: 1.333–1.571]
Axilla PAT	1.776 (0.47) [95% CI: 1.490–2.062]	Dorsum Hand PAT	2.042 (0.70) [95% CI: 1.647–2.437]
Axilla	1.607 (0.33) [95% CI: 1.408–1.806]	Dorsum Hand	1.539 (0.30) [95% CI: 1.365–1.714]

**Table 9:** Ultrasound measurements of subcutaneous tissue thickness in the arm and forearm, expressed in millimeters. The segment highlighted in green indicates a statistically significant difference in thickness between the affected and healthy limbs. This highlights the specific level/region where lymphedema has caused notable subcutaneous tissue alterations. SD: standard deviation. CI: confidence interval. The segment highlighted in green indicates a statistically significant difference in subcutaneous tissue thickness between the affected and healthy limbs for the arm.

Subcutaneous Tissue			
Arm	Mean (SD) [95% CI]	Forearm	Mean (SD) [95% CI]
Ant 1 PAT	6.284 (3.30) [95% CI: 4.386–8.182]	Ant 1 PAT	8.211 (2.24) [95% CI: 6.919–9.503]
Ant 1	7.079 (3.10) [95% CI: 5.296–8.861]	Ant 1	6.163 (1.95) [95% CI: 5.039–7.286]
Ant 2 PAT	8.262 (3.20) [95% CI: 6.428–10.10]	Ant 2 PAT	7.174 (2.313) [95% CI: 5.838–8.509]
Ant 2	6.616 (1.94) [95% CI: 5.492–7.741]	Ant 2	4.006 (2.10) [95% CI: 3.212–6.086]
Post 1 PAT	14.44 (5.11) [95% CI: 11.49–17.39]	Post 1 PAT	7.139 (3.453) [95% CI: 5.145–9.132]
Post 1	13.36 (5.63) [95% CI: 11.622–12.267]	Post 1	13.35 (6.156) [95% CI: 9.792–16.90]
Post 2 PAT	13.21 (4.52) [95% CI: 10.60–15.82]	Post 2 PAT	7.546 (3.135) [95% CI: 5.736–9.356]
Post 2	6.271 (2.29) [95% CI: 11.30–17.14]	Post 2	5.686 (2.214) [95% CI: 4.408–6.964]
Axilla PAT	6.546 (3.027) [95% CI: 4.479–7.929]	Dorsum Hand PAT	3.349 (1.486) [95% CI: 2.490–4.207]
Axilla	5.784 (2.782) [95% CI: 4.398–7.683]	Dorsum Hand	2.005 (0.433) [95% CI: 1.688–2.330]

**Table 10:** Ultrasound measurements of the superficial fascia thickness in the arm and the forearm, expressed in millimeters. SD: standard deviation. CI: confidence interval.

Superficial Fascia			
Arm	Mean (SD) [95% CI]	Forearm	Mean (SD) [95% CI]
Ant 1 PAT	0.2843 (0.06) [95% CI: 0.2530–0.3156]	Ant 1 PAT	0.2800 (0.06) [95% CI: 0.2480–0.3120]
Ant 1	0.2757 (0.04) [95% CI: 0.2512–0.3002]	Ant 1	0.2871 (0.05) [95% CI: 0.2593–0.3150]
Ant 2 PAT	0.2936 (0.06) [95% CI: 0.2617–0.3255]	Ant 2 PAT	0.2514 (0.05) [95% CI: 0.2256–0.2772]
Ant 2	0.2679 (0.06) [95% CI: 0.2337–0.3020]	Ant 2	0.2843 (0.06) [95% CI: 0.2521–0.3165]
Post 1 PAT	0.2700 (0.06) [95% CI: 0.2350–0.3050]	Post 1 PAT	0.3143 (0.06) [95% CI: 0.2794–0.3491]
Post 1	0.2745 (0.07) [95% CI: 0.2303–0.3188]	Post 1	0.3150 (0.05) [95% CI: 0.2858–0.3442]
Post 2 PAT	0.3257 (0.06) [95% CI: 0.2892–0.3622]	Post 2 PAT	0.2686 (0.04) [95% CI: 0.2461–0.2911]
Post 2	0.3650 (0.10) [95% CI: 0.3088–0.4212]	Post 2	0.2800 (0.03) [95% CI: 0.2604–0.2996]
Axilla PAT	0.2814 (0.06) [95% CI: 0.2567–0.3217]	Dorsum Hand PAT	0.2814 (0.06) [95% CI: 0.2456–0.3172]
Axilla	0.2929 (0.05) [95% CI: 0.2728–0.3395]	Dorsum Hand	0.2929 (0.05) [95% CI: 0.2654–0.3204]

**Table 11:** Ultrasound measurements of deep fascia thickness in the arm and forearm expressed in millimeters. SD: standard deviation. CI: confidence interval.

Deep Fascia			
Arm	Mean (SD) [95% CI]	Forearm	Mean (SD) [95% CI]
Ant 1 PAT	0.428 (0.06) [95% CI: 0.3933–0.4624]	Ant 1 PAT	0.443 (0.10) [95% CI: 0.3840–0.5017]
Ant 1	0.443 (0.07) [95% CI: 0.4015–0.4842]	Ant 1	0.424 (0.10) [95% CI: 0.3839–0.4632]
Ant 2 PAT	0.452 (0.11) [95% CI: 0.3866–0.5177]	Ant 2 PAT	0.376 (0.11) [95% CI: 0.3153–0.4376]
Ant 2	0.502 (0.11) [95% CI: 0.4403–0.5640]	Ant 2	0.413 (0.10) [95% CI: 0.3790–0.4467]
Post 1 PAT	0.402 (0.11) [95% CI: 0.3351–0.4695]	Post 1 PAT	0.501 (0.13) [95% CI: 0.4268–0.5761]
Post 1	0.383 (0.11) [95% CI: 0.3100–0.4555]	Post 1	0.53 (0.14) [95% CI: 0.4485–0.6115]
Post 2 PAT	0.551 (0.20) [95% CI: 0.4656–0.6358]	Post 2 PAT	0.429 (0.10) [95% CI: 0.3691–0.4881]
Post 2	0.514 (0.11) [95% CI: 0.4531–0.5755]	Post 2	0.464 (0.13) [95% CI: 0.3915–0.5357]
Axilla PAT	0.423 (0.10) [95% CI: 0.3688–0.4774]	Dorsum Hand PAT	0.441 (0.12) [95% CI: 0.3661–0.5155]
Axilla	0.439 (0.11) [95% CI: 0.3752–0.5033]	Dorsum Hand	0.449 (0.10) [95% CI: 0.3875–0.5096]

**Table 12:** Echogenicity of the subcutaneous tissue in the arm and axilla, and in the forearm and dorsum of hand expressed on a grayscale ranging from 0 (black) to 255 (white), provides a quantitative measure of tissue density. This scale helps to assess variations in tissue composition, with higher values indicating greater echogenicity, potentially reflecting structural changes such as fibrosis or fluid accumulation in the context of lymphedema. The segment highlighted in green indicates a statistically significant difference in echogenicity between the affected and healthy limbs.

Subcutaneous Tissue			
Arm	Mean (SD) [95% CI]	Forearm	Mean (SD) [95% CI]
Ant 1 PAT	55.48 (13.92) [95% CI: 47.44–63.52]	Ant 1 PAT	71.19 (13.59) [95% CI: 63.34–79.03]
Ant 1	53.7 (9.11) [95% CI: 48.44–58.96]	Ant 1	53.1 (9.77) [95% CI: 47.49–58.71]
Ant 2 PAT	66.62 (14.64) [95% CI: 58.17–75.07]	Ant 2 PAT	65.18 (15.56) [95% CI: 56.19–74.16]
Ant 2	57.4 (9.86) [95% CI: 51.70–63.09]	Ant 2	56.12 (11.04) [95% CI: 49.75–62.50]
Post 1 PAT	54.09 (14.35) [95% CI: 45.80–62.38]	Post 1 PAT	62.98 (7.81) [95% CI: 58.47–67.49]
Post 1	60.11 (16.08) [95% CI: 50.82–69.39]	Post 1	56.35 (14.52) [95% CI: 47.97–64.74]
Post 2 PAT	61.19 (14.97) [95% CI: 52.55–69.83]	Post 2 PAT	62.13 (17.69) [95% CI: 51.91–72.34]
Post 2	60.9 (11.41) [95% CI: 54.32–67.49]	Post 2	54.2 (18.01) [95% CI: 43.81–64.60]
Axilla PAT	48.24 (11.74) [95% CI: 42.46–57.12]	Dorsum Hand PAT	58.88 (20.85) [95% CI: 46.84–70.92]
Axilla	45.79 (10.95) [95% CI: 40.38–54.65]	Dorsum Hand	56.43 (13.34) [95% CI: 46.42–63.30]

**Table 13:** Correlation between the total CLUE score and subcutaneous tissue echogenicity highlights a relationship where higher CLUE scores are associated with increased echogenicity.

	Arm Post 1	Forearm Post 1	Forearm Post 2	Dorsum Hand
Pearson r (r)	0.6322	0.6309	0.6472	0.6359
*p* value (two-tailed)	0.0153	0.0155	0.0123	0.0145
Significant? (alpha = 0.05)	Yes	Yes	Yes	Yes

**Table 14:** Correlation between the volume of the affected limb and the thickness of the subcutaneous tissue highlights a significant relationship. As the limb’s volume increases, particularly in lymphedema cases, there is a proportional thickening of the subcutaneous tissue, especially in key regions such as the distal posterior arm and posterior forearm.

	Arm Post 1	Forearm Post 1	Forearm Post 2
Pearson r (r)	0.5386	0.7015	0.6368
*p* value (two-tailed)	0.0469	0.0052	0.0143
Significant? (alpha = 0.05)	Yes	Yes	Yes

**Table 15:** Correlation between the duration of lymphedema and subcutaneous tissue/echogenicity reveals distinct patterns. Over time, there is a positive correlation between lymphedema duration and increased subcutaneous tissue thickness in the distal anterior forearm. Conversely, there is a negative correlation with echogenicity in the distal posterior forearm. This suggests that longer lymphedema duration leads to subcutaneous thickening in the anterior regions, while a decrease in posterior echogenicity may be linked to the delayed lymph accumulation in these areas, reflecting the disease’s progression and impact on tissue structure.

	Thickness Subcutaneous Tissue Forearm Ant 2	Echogenicity Subcutaneous Tissue Forearm Post 2
Pearson r (r)	0.625	−0.5475
*p* value (two-tailed)	0.0168	0.0427
Significant? (alpha = 0.05)	Yes	Yes

**Table 16:** Intra-rater reliability of the US thickness measurements of skin, superficial fascia, deep fascia, and subcutaneous tissue.

Region/Level	ICC
Arm Ant 1	0.92 (0.88–0.96)
Arm Ant 2	0.92 (0.88–0.96)
Arm Post 1	0.94 (0.90–0.98)
Arm Post 2	0.92 (0.88–0.96)
Axilla	0.92 (0.88–0.96)
Forearm Ant 1	0.92 (0.88–0.96)
Forearm Ant 2	0.92 (0.88–0.96)
Forearm Post 1	0.92 (0.88–0.96)
Forearm Post 2	0.92 (0.88–0.96)
Dorsum of Hand	0.88 (0.85–0.90)

## Data Availability

The data presented in this study are available upon request from the corresponding author. The data are not publicly available due to privacy reasons.
